# Whole-Genome Sequences of Low-Virulence Strain CB3 and Mild Strain CB7 of *Chlamydia psittaci*

**DOI:** 10.1128/genomeA.00456-14

**Published:** 2014-06-05

**Authors:** Jun Chu, Ruixue Sun, Zongxue Wu, Shanshan Liu, Dongfang Li, Qiang Zhang, Yong Ling, Yanping Gong, Renhua Wu, Honglong Wu, Jizhang Zhou, Cheng He, Peixiang Ni

**Affiliations:** a Key Laboratory of Animal Epidemiology and Zoonosis of Ministry of Agriculture, College of Veterinary Medicine, China Agriculture University, Beijing, China; b BGI-Tianjin, Tianjin, China; c Tianjin Medical Genomics Technology Engineering Center, Tianjin, China; d Lanzhou Veterinary Research Institute, Chinese Academy of Agricultural Sciences, Lanzhou, China

## Abstract

Avian *Chlamydia psittaci* is an obligate intracellular zoonotic pathogen especially dispersed from birds, and it is known to cause pericarditis, pneumonia, lateral nasal adenitis, peritonitis, hepatitis, splenitis, and other diseases. Generalized infections result in fever, anorexia, lethargy, and diarrhea, depending on the chlamydial genotype and the affected bird species. Although many complete genomes of *C. psittaci* have been sequenced, we report here the genomes of two strains isolated from the free-living sparrows (strain CB3) and vinous-throated parrotbill (strain CB7) in China, which were first isolated from the spleens of healthy birds in a routine investigation.

## GENOME ANNOUNCEMENT

Avian chlamydial strains can cause serious illness and possibly death in humans (1). The disease in ducks and turkeys is of particular concern, as transmission to humans is common during the handling and slaughter of the birds (2, 3). Free-living wild birds act as a reservoir for *Chlamydia psittaci* and pose a threat pf transmission to farm poultry (4). Although several whole-genome sequences of *C. psittaci* originating from domestic birds have been recorded (5–7), we report here the genome sequences and annotations of two strains of *C. psittaci* isolated from the spleens of sparrows (CB3) and the vinous-throated parrotbill (CB7) (8, 9). *C. psittaci* CB3 was isolated in embryonated chicken eggs and identified using Gimenez stain and immunofluorescence stain (DakoCytomation, United Kingdom). Subsequently, it was determined in a mouse model to be an avirulent bacterial strain (8), and it belongs to a low-virulence strain in 2-week-old specific-pathogen-free (SPF) chickens (9). Meanwhile, *C. psittaci* CB7 was also isolated and identified as previously described (8). In an SPF pathogenesis model, it is confirmed to be a mild strain compared to *C. psittaci* 6BC (9). Moreover, the obtained sequences for detecting the *ompA* restriction map were identical and clustered within genotype A, as described by Denamur et al. (10).

These two genomes were determined by the whole-genome shotgun strategy using the Illumina sequencing technology. The reads from the host were filtered before processing with the following analysis. *De novo* assemblies were performed with the software program SOAP*denovo* (version 1.05). The genome sequences of these two strains were 1,171,837 bp and 1,189,806 bp in size, respectively. The CB3 strain was assembled in one scaffold, and 19 scaffolds were assembled for the CB7 strain, with an *N*_50_ size of 786,985 bp. The G+C contents of these two genomes are 39.06% and 39.03%, respectively, which are similar to those of other avian *Chlamydiaceae* (5, 7). In a comparison with the 6BC strain, a scaffold with length 7,577 bp in the CB7 strain was identified to be a plasmid sequence.

Each genome was annotated by an in-house pipeline. A total of 1,004 and 1,009 putative coding sequences in the CB3 and CB7 chromosomes were predicted by the Glimmer3 software package, respectively. Also, 8 putative coding sequences were identified in the plasmid sequence of CB7. Each strain had one rRNA operon and 38 tRNAs by RNAmmer and tRNAscan-SE, respectively (11). The molecular characterization of the two isolates by constructing a phylogenetic tree with single nucleotide polymorphisms based on those released public genome sequences revealed that they belong to genotype A.

### Nucleotide sequence accession numbers.

This whole-genome shotgun project of strain CB7 has been deposited at DDBJ/EMBL/GenBank under the accession no. JMBZ00000000 (version JMBZ01000000), and strain CB3 under the accession no. JMEI00000000 (version JMEI01000000).
